# RBMX Protein Expression in T-Cell Lymphomas Predicts Chemotherapy Response and Prognosis

**DOI:** 10.3390/cancers13194788

**Published:** 2021-09-24

**Authors:** Franziska Lea Schümann, Marcus Bauer, Elisabeth Groß, Denis Terziev, Andreas Wienke, Claudia Wickenhauser, Mascha Binder, Thomas Weber

**Affiliations:** 1 Department of Internal Medicine IV, Haematology and Oncology, University Hospital Halle (Saale), Martin-Luther-University Halle-Wittenberg, 06120 Halle, Germany; franziska.schuemann@uk-halle.de (F.L.S.); elisabeth.gross@uk-halle.de (E.G.); denis.terziev@uk-halle.de (D.T.); mascha.binder@uk-halle.de (M.B.); 2 Institute of Pathology, University Hospital Halle (Saale), Martin-Luther-University Halle-Wittenberg, 06112 Halle, Germany; marcus.bauer@uk-halle.de (M.B.); claudia.wickenhauser@uk-halle.de (C.W.); 3 Institute of Medical Epidemiology, Biometrics and Informatics, Martin-Luther-University Halle-Wittenberg, 06112 Halle, Germany; andreas.wienke@uk-halle.de

**Keywords:** T-cell non-Hodgkin’s lymphomas, PTCL, RBMX, hnRNP G, drug resistance, immunohistochemistry

## Abstract

**Simple Summary:**

Patients with T-cell non-Hodgkin’s lymphomas (T-NHL) are often chemotherapy refractory and subsequently have poor prognosis. So far, mechanisms leading to this primary chemotherapy refractoriness and factors identifying such cases are not well established. This study investigated the prognostic relevance of the RNA binding protein X (RBMX) in 53 T-NHL cases using conventional immunohistochemistry. As shown, low RBMX expression was associated with better response to anthracycline-containing first-line treatment. Furthermore, low RBMX expression predicted an improved overall survival (OS) and progression-free survival (PFS). These results suggest that RBMX protein expression levels might be a contributing factor towards chemotherapy resistance and thus affect prognosis of patients with T-cell lymphomas.

**Abstract:**

T-cell non-Hodgkin’s lymphomas (T-NHL) are a heterogeneous group of lymphomas with a mature T-cell phenotype. While in some hematological diseases the prognosis improved over the last decades, T-NHL cases often relapse early or present with an initially refractory course. Recently, it has been shown that RNA binding proteins have a crucial role for malignant tumor initiation, progression and treatment response while contributing to chemotherapy resistance. Therefore, we investigated the protein expression of the RNA binding protein X (RBMX), which has been shown to be of great relevance in disease initiation and progression in hematological diseases in 53 T-NHL cases using conventional immunohistochemistry. Low RBMX expression was associated with better response to anthracycline-containing first-line treatment. Furthermore, low RBMX expression predicted an improved overall survival and progression-free survival in univariate analysis. Multivariable Cox regression revealed RBMX as an independent prognostic marker for overall survival (*p* = 0.007; hazard ratio (HR) = 0.204; 95% confidence interval (CI): 0.064–0.646) and progression-free survival (*p* = 0.006; HR = 0.235; 95% CI: 0.083–0.666). The study identifies low RBMX expression to predict better chemotherapy response, overall survival and progression-free survival in patients with T-cell non-Hodgkin’s lymphomas. These results suggest that RBMX protein expression levels might be a contributing factor towards chemotherapy resistance and thus affect prognosis. Hence, RBMX may be a potential therapeutic target and prognostic marker in T-cell lymphomas.

## 1. Introduction

T-cell non-Hodgkin’s lymphomas (T-NHL) arise from post-thymic lymphocytes and represent 10–15% of all non-Hodgkin’s lymphomas in western countries [1]. T-NHL are highly heterogeneous in their clinical presentation, histologic features, and pathogenesis [2]. The disease is characterized by frequent relapse, and an initially refractory course is not uncommon. For most subtypes, the first-line treatment regime is typically an anthracycline-containing chemotherapy combination, such as CHO(E)P (cyclophosphamide, doxorubicin, vincristine, (etoposide), and prednisone) [3]. Approximately 30% of patients face primary refractory disease [4,5], and most patients with refractory or relapsed T-NHL have poor outcomes with short survival [6]. Therefore, it urgently requires further research for precise prognostic indicators and novel treatment options in order to improve the survival of affected patients.

Recently, it has been reported that RNA-binding proteins play an important role in cancer progression, and even contribute to chemotherapy resistance [7]. Heterogeneous nuclear ribonucleoproteins (hnRNPs) are a large family of chromatin-associated RNA-binding proteins with more than 30 different members. Among these, the X-linked RNA-binding motif protein (RBMX, also named hnRNP G) is one of the least characterized proteins concerning its biological functions [8]. RBMX was originally recognized as a nuclear protein that is part of the supraspliceosome, where it regulates alternative splice site selection depending on its concentration [9,10]. RBMX is also known to regulate the proper cohesion of sister chromatids during cell division [11]. Recently, several studies have reported replication defects in cells lacking RBMX, and the authors pointed to a key role of RBMX in genome stability [12,13].

The function of RBMX during carcinogenesis and promoting therapy resistance has only sporadically been studied and so far has remained insufficiently understood. RBMX has been proposed as a potential tumor suppressor in several cancer types, including oral squamous carcinoma [8,14,15] and lung cancer [16]. However, reports of RBMX expression levels in cancer samples are more contradictory. Low RBMX expression levels have been associated with poor outcome in endometrial cancer [17,18] and bladder cancer [19]. In contrast, recent studies interestingly show poor outcome in hepatocellular carcinomas (HCC) [7] as well as head and neck cancers [20] when RBMX was highly expressed. Furthermore, RBMX was overexpressed in individuals with acute myeloid leukemia (AML) compared to healthy ones, and loss of RBMX delayed leukemia development [21].

In addition, RBMX was intimately involved in chemoresistance according to previous studies. In 2012, it was shown by Adamson et al. that the depletion of RBMX sensitizes cells to DNA damage caused by ionizing radiation and several genotoxic drugs (mitomycin C, chlorambucil, oxaliplatin, and carboplatin). Moreover, exome sequencing analysis show mutations in the RBMX gene in vemurafenib-resistant thyroid carcinoma cells [22]. On the contrary, it has recently been shown that in HCC cells, sorafenib resistance was increased when RBMX was overexpressed [7]. To further understand the role of RBMX in chemotherapy resistance and cancer prognosis of T-cell lymphomas, the present study investigates the immunohistochemical protein expression of RBMX in combination with the clinical outcomes in patients with T-NHL.

## 2. Materials and Methods

### 2.1. Patients and Tissue Samples

This is a retrospective single-center analysis of 53 patients forming a convenience sample who were treated between 2006 and March 2020 at the University Hospital Halle (Saale). Only patients with available formalin-fixed, paraffin-embedded (FFPE) T-cell leukemia and lymphoma tissues were included. The tissue samples originated from 43 patients that had been integrated into a tissue microarray (TMA) for a previous study and from 10 large tissue sections obtained for this study. Patients were identified by a review of the internal hospital database records. Patients were included in the analysis if they were > 18 years of age with a biopsy-proven diagnosis of T-NHL according to the WHO classification. All samples were histopathological reviewed by two pathologists (C.W. and M.B.) to verify the diagnosis according to WHO criteria 2017 [2]. Five samples were excluded since the integrated tissue samples did not originate from the primary diagnosis or the diagnosis was not confirmed. Clinicopathological characteristics at the time of primary diagnosis, including age, sex, histological phenotypes, B symptoms, Ann Arbor stage, international prognostic index (IPI), Eastern Cooperative Oncology Group (ECOG) status, bone marrow involvement (BMI), lactate dehydrogenase (LDH) level, white blood cell (WBC) count and Ki-67 expression along with treatment regime, chemotherapy response, the occurrence of relapses, and follow-up data were recorded in the TMA cohort (*n* = 43). Chemotherapy resistance was defined as stable disease (SD) or progressive disease (PD) after first-line treatment, while chemosensitive patients had a complete response (CR) or a partial response (PR) according to RECIST [23]. In accordance with the Declaration of Helsinki, this study was performed and approved by the Ethics Committee at the Martin-Luther-University of Halle-Wittenberg (#2020–033).

### 2.2. Tissue Microarray Construction

FFPE leukemia and lymphoma tissues from 53 non-selected patients were obtained from the Institute of Pathology, University Hospital Halle (Saale). Tissue microarrays (TMAs) containing two 0.6 mm tissue cylinders of each donor block were constructed using a manual tissue arrayer (Beecher Instruments Inc., Sun Prairie, WI, USA). A slide stained with hematoxylin and eosin (H&E) was prepared from each donor block and representative tumor regions were morphologically identified and marked on each slide by a pathologist (M.B). From these defined areas, two tissue cores with a diameter of 0.6 mm were taken and arrayed on a recipient paraffin block. Adequate control tissues, including liver tissue, tonsil tissue, breast carcinoma, seminoma, prostate carcinoma, and osteosarcoma for specific antibodies were also included.

### 2.3. Immunohistochemistry

Immunohistochemistry (IHC) analysis was performed on a Bond III automated immunostainer (Leica Biosystems Nussloch GmbH, Wetzlar, Germany) using the Bond Polymer Refine Detection Kit (DS9800-CN). The primary antibody for RBMX (1:150; Abcam, Cambridge, UK; ab190352) was applied as recommended by the manufacturer. Immunostaining was assessed by two investigators (M.B. and F.L.S.) blinded to additional pathological and clinical data using Zeiss Axioscope 5 microscope (Carl Zeiss Microscopy GmbH, Jena, Germany). Semi-quantitative H-scoring [24] was performed in adherence to the following steps: Brown immunoreactivity of cell nuclei was taken as positive and the proportion of negative cells (P0) as well as staining at low (P1), moderate (P2), or high (P3) levels of intensity were scored. The H-score for one patient (H-score = (% of cells stained at intensity category 1 × 1) + (% of cells stained at intensity category 2 × 2) + (% of cells stained at intensity category 3 × 3)) was calculated from the mean of two stains. The conclusions of the two inspectors were in complete agreement in approximately 88% of the cases confirming this scoring method as reproducible. In addition, 10 normal lymph node tissues were stained to compare the RBMX expression levels of healthy and tumor tissues.

### 2.4. Survival and Statistical Analysis

IBM SPSS statistic software, version 27.0, for Mac (International Business Machines Corporation, Armonk, NY, USA) was used for data analysis. All analyses were performed for T-NHL overall, followed by nodal T-NHL phenotypes. Comparison of continuous variables between two groups was computed with unpaired *t*-tests and between multiple groups with one-way ANOVA with Bonferroni’s correction. Overall survival (OS) and progression-free survival (PFS) rates were obtained using the Kaplan−Meier method. Statistical comparisons between groups were made by log rank tests. Receiver operating characteristic (ROC)-analysis was used to determine a cutoff value for RBMX expression to divide the samples into two groups: RBMX^high^ and RBMX^low^. The resulting cutoff point was an H-score of 175. Additionally, ROC was used to evaluate RBMX as a predictive factor for chemotherapy response. Multivariable analysis was performed using a Cox proportional hazards model to assess the independent effect of prognostic variables on PFS and OS. OS was defined as the time from primary diagnosis until last follow-up or death from any cause. PFS was defined as the time from primary diagnosis until lymphoma progression or death from any cause. Patients alive at the last follow-up date were censored. All *p*-values were interpreted exploratorily.

## 3. Results

### 3.1. Patient and Treatment Characteristics

A total of 43 patients with T-NHL fulfilled the inclusion criteria and had available clinical data (TMA cohort). In 10 cases, only the H-score was available but not the clinical data. The median age of all the evaluated patients was 66 years (range, 36–92 years), with a male-to-female ratio of 2.1:1. At the time of analysis, median follow-up time for living patients was 25.0 months (range, 0 to 142). Overall, 20 patients (46.5%) had died. The study included 19 cases of peripheral T-cell lymphomas with T-helper phenotype (angioimmunoblastic T-cell lymphoma (AITL)) and nodal peripheral T-cell lymphoma with T follicular helper phenotype (PTCL-TFH)), eight cases of peripheral T-cell lymphomas, not otherwise specified (PTCL-NOS), seven cases of anaplastic large-cell lymphomas, ALK-negative (ALCL, ALK-negative), 12 cases of intestinal T-NHL, two cases of extranodal natural killer/T-cell lymphoma, nasal type (NKTCL) and five cases of other subtypes (Mycosis fungoides (MF) *n* = 1, Sézary syndrome (SS) *n* = 1, subcutaneous panniculitis-like T-cell lymphoma (SPTCL) *n* = 1, T-cell prolymphocytic leukemia (T-PLL) *n* = 1, and T-cell large granular lymphocytic leukemia (T-LGL) *n* = 2). The recorded clinicopathologic characteristics are summarized in Table 1.

### 3.2. Expression of RBMX in T-Cell Lymphomas

**Figure 1:**
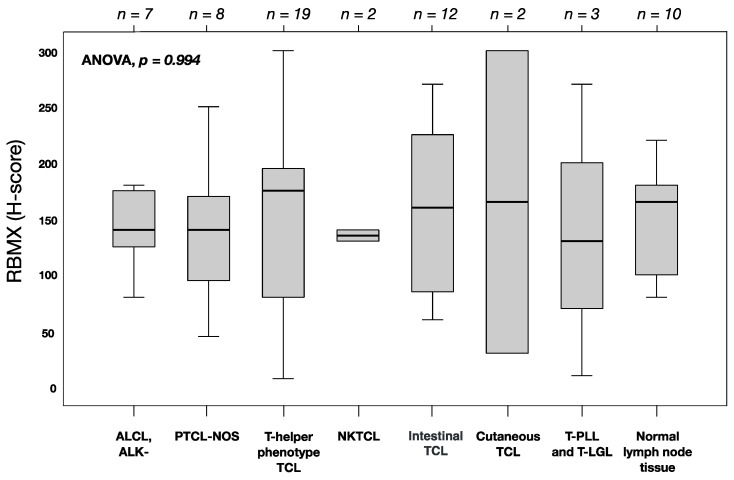
RBMX expression in T-cell lymphoma and non-tumor tissues analyzed by immunohistochemistry. Boxplots represent the median and interquartile range of protein levels. *p*-values were calculated using ANOVA and post-hoc tests for comparison between histological phenotypes and non-tumor tissues. The number of tissue samples is shown at top. Abbreviations: ALCL, ALK-: anaplastic large-cell lymphoma, ALK-negative; TCL: T-cell lymphoma; PTCL-NOS: peripheral T-cell lymphoma, not otherwise specified; NKTCL: extranodal natural killer/T-cell lymphoma, nasal type; T-PLL: T-cell prolymphocytic leukemia; T-LGL: T-cell large granular lymphocytic leukemia.

**Figure 2:**
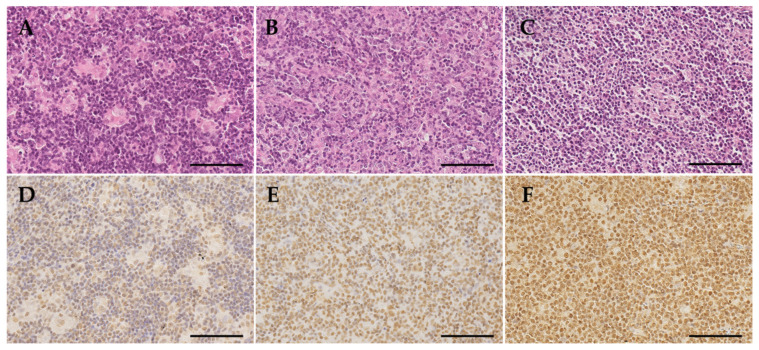
Histomorphology and RBMX immunohistochemistry. All samples were stained with hematoxylin and eosin (H&E) as exemplary shown in micrographies (**A**–**C**). The immunohistochemical stains for RBMX were examined and evaluated in conjunction with the H&E stains. Immunohistochemical staining showing low (**D**), middle (**E**), and high (**F**) levels of nuclear RBMX expression. Original magnification ×40, the scale bars are 100 µm.

IHC for RBMX was analyzed on a total of 53 T-cell lymphomas and ten normal non-tumor lymph nodes using FFPE tissues. The overall mean of RBMX protein expression (H-score) was 145 (standard deviation (SD) = 68). No relevant differences in the RBMX expression levels among the histological phenotypes or compared to normal lymph nodes were observed (ANOVA; *p* = 0.994; Figure 1). Representative immunohistochemical staining showing different levels of nuclear RBMX expression are illustrated in Figure 2.

### 3.3. Association between RBMX Expression and Clinicopathological Characteristics

We investigated the association between nuclear RBMX protein expression and clinicopathological characteristics including sex, age, B symptoms, Ann Arbor stage, IPI, BMI, ECOG status, WBC, LDH, Ki-67 expression, the occurrence of relapses, and response to first-line chemotherapy (regardless of the regime used). The results of unpaired t-tests are summarized in Table 2. In patients with T-NHL, high RBMX expression was associated with a normal white blood cell count (WBC normal vs. upper limit of normal: mean 193 vs. 118; *p* = 0.023) and non-response to first-line chemotherapy (resistant vs. sensitive: mean 178 vs. 128; *p* = 0.029). Furthermore, no strong association between high RBMX expression and the absence of bone marrow involvement (noBMI) (noBMI vs. BMI: mean 152 vs. 107; *p* = 0.090) was observed. No relevance with sex, age, B symptoms, Ann Arbor stage, IPI, ECOG status, LDH level, Ki-67 expression, and the occurrence of relapses was noted in T-NHL. In patients with nodal T-NHL, high RBMX expression was associated with noBMI (noBMI vs. BMI: mean 153 vs. 94; *p* = 0.034). In addition, no strong associations between high RBMX expression with a normal white blood cell count (WBC normal vs. upper limit of normal: mean 186 vs. 126; *p* = 0.089) and non-response to first-line chemotherapy (resistant vs. sensitive: mean 167 vs. 130; *p* = 0.119) were observed. No relevant association with sex, age, B symptoms, Ann Arbor stage, IPI, ECOG status, LDH level, Ki-67 expression, and the occurrence of relapses was noted in nodal T-NHL.

### 3.4. Predictive Value of RBMX Expression to Anthracycline-Containing First-Line Treatment

**Figure 3:**
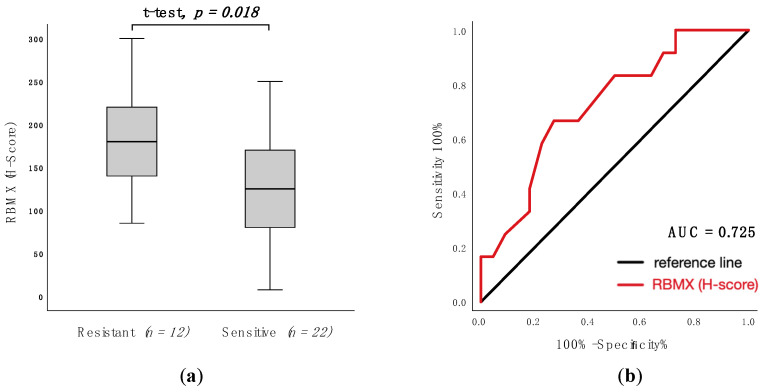
RBMX expression according to response following anthracycline-containing first-line treatment in T-NHL. (**a**): Corresponding histogram of RBMX expression (H-score) in resistant and Scheme 0. (**b**): ROC analysis of RBMX expression (H-score) to discriminate between resistant and sensitive groups. AUC (area under the curve) = 0.725 (95% confidence interval: 0.550–0.901; *p* = 0.012). Abbreviations: CHOP: cyclophosphamide, doxorubicin, vincristine, and prednisone content chemotherapy; CHOEP: cyclophosphamide, doxorubicin, vincristine, etoposide, and prednisone content chemotherapy.

To assess RBMX expression as a predictive factor for response to anthracycline-containing first-line treatment, ROC-analysis and t-tests were used. In patients with T-NHL, high RBMX expression was associated with non-response (resistant vs. sensitive: mean 185 vs. 127; *p* = 0.018; Figure 3a) and the area under the curve (AUC) was 0.725 (95% CI: 0.550–0.901; *p* = 0.012; Figure 3b) (specificity 66.7%, sensitivity 72.7%). In nodal T-NHL, high RBMX expression was not strongly associated with non-response (resistant vs. sensitive: mean 169 vs. 130; *p* = 0.120). The AUC was 0.662 (95% CI: 0.463–0.861; *p* = 0.111) (specificity 60.0%, sensitivity 71.4%).

### 3.5. RBMX Expression Predicts OS and PFS in T-Cell Lymphomas

**Figure 4:**
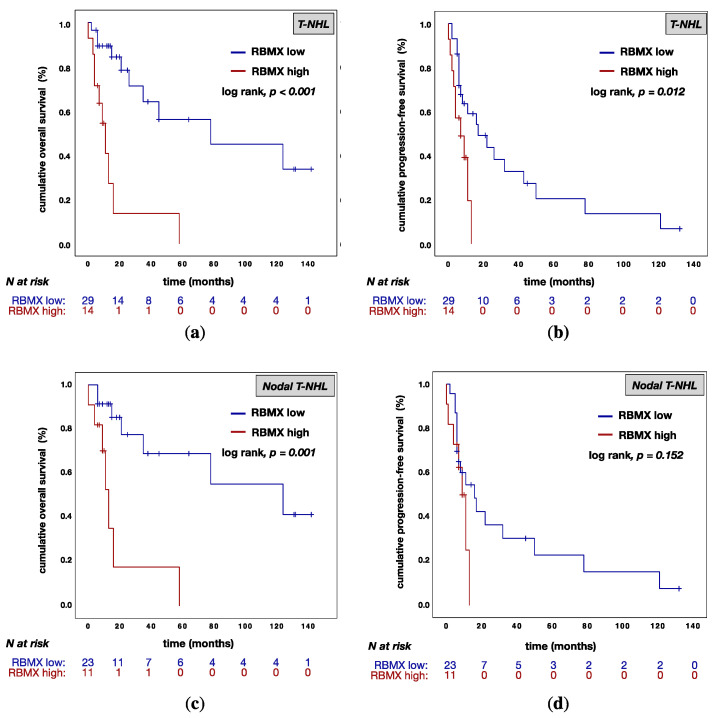
Kaplan−Meier (KM) curves for overall survival (OS) and progression-free survival (PFS) according to RBMX expression. (**a**): KM curve for OS according to RBMX expression in T-NHL; (**b**): KM curve for PFS according to RBMX expression in T-NHL; (**c**): KM curve for OS according to RBMX expression in nodal T-NHL: (**d**) KM curve of PFS according to RBMX expression in nodal T-NHL. Abbreviations: T-NHL: T-cell non-Hodgkin’s lymphomas; RBMX low: RBMX expression with an H-score below 175; RBMX high: RBMX expression above or equal to an H-score of 175; *N*: number of patients.

In univariate analysis, RBMX^high^ expression was associated with a poor OS rate in T-NHL (RBMX^low^ vs. RBMX^high^: median OS 78.0 (95% CI: 0.0–160.2) vs. 11.0 (95% CI: 5.5–16.5) months; *p* < 0.001; Figure 4a). This finding agrees with our observations in the nodal T-NHL subtypes (RBMX^low^ vs. RBMX^high^: median OS 124.0 (95% CI: 14.0–233.9) vs. 13.0 (95% CI: 8.7–17.3) months; *p* = 0.001; Figure 4c). Furthermore, the RBMX^high^ expression was associated with a poor PFS rate in patients with T-NHL (RBMX^low^ vs. RBMX^high^: median OS 17.0 (95% CI: 2.6–31.4) vs. 7.0 (95% CI: 0.0–14.4) months; *p* = 0.012; Figure 4b). In nodal T-NHL, RBMX expression was not significantly associated with a poor PFS (RBMX^low^ vs. RBMX^high^: median OS 16.0 (95% CI: 4.8–27.2) vs. 9.0 (95% CI: 5.6–12.4) months; *p* = 0.152; Figure 4d).

Multivariable analysis of age, sex, B symptoms, BMI, Ann Arbor stage (only for nodal T-NHL), and RBMX expression was performed for OS and PFS. Table 3 summarizes the results of all tested variables. It turned out that RBMX expression was associated with a poor OS (*p* = 0.007; HR = 0.204; 95% CI: 0.064–0.646; Table 3) in T-NHL. In addition, RBMX expression (*p* = 0.006; HR = 0.235; 95% CI: 0.083–0.666; Table 3) and BMI (*p* = 0.004; HR = 0.243; 95% CI: 0.094–0.628; Table 3) were associated with a poor PFS in T-NHL. In patients with T-NHL, B symptoms (*p* = 0.087; HR = 0.466; 95% CI: 0.194–1.118; Table 3) were not strongly associated with poor PFS. In patients with nodal T-NHL phenotypes the RBMX expression was an independent prognostic marker only for OS (*p* = 0.038; HR = 0.149; 95% CI: 0.025–0.898).

## 4. Discussion

To the best of our knowledge, we present the first analysis of the prognostic impact of RBMX protein expression in patients with T-cell lymphomas. RBMX (also called hnRNP G) is an RNA-binding-motif gene located on the X-chromosome. Beyond its function as a splicing factor, RBMX plays an important role in DNA-damage protection [25], chromosome segregation [11], and genome stability [12,13]. Still, the function of RBMX during carcinogenesis constitutes a desideratum of research. In this study, we investigated the immunohistochemical expression of RBMX in combination with the clinical outcomes in 53 patients with T-cell non-Hodgkin’s lymphomas.

RBMX was expressed homogeneously across the histological phenotypes. RBMX expression was examined in normal non-tumor lymph node tissue and T-cell lymphoma, showing lower expression in healthy tissues compared to tumor samples with high RBMX expression (non-tumor vs. RBMX^high^: mean 150 (SD = 49) vs. 220 (SD = 44); *p* = 0.024). Subsequently, we assessed the association of RBMX expression and clinicopathological characteristics at the time of primary diagnosis. Interestingly, high RBMX expression was associated with markers that are known for moderate disease progression (normal WBC count, noBMI). In contrast, high RBMX expression was associated with resistance to first-line treatment, regardless of the regime used. Due to the functions of RBMX presented above, we examine the hypothesis that the protein might be contributing to drug resistance. Previously, several studies investigated the association between RBMX expression and drug resistance, pointing out that high expression levels led to non-response to various drugs [7]. We investigated the correlation between RBMX expression and the response to anthracycline-containing first-line treatment. In agreement with the previous reports, low RBMX expression levels predict better response to anthracycline-containing chemotherapy in patients with T-NHL (t-test *p*-value = 0.018; ROC-analysis AUC = 0.725). Former studies indicated an opposite association of RBMX expression levels and prognosis in different cancer types [7,17,18,20,21]. In our univariate analysis, low RBMX levels predict improved overall survival in patients with T-NHL (median OS 78.0 vs. 11.0 months; *p* < 0.001) and nodal T-NHL (median OS 124.0 vs. 13.0 months; *p* = 0.001) and also better progression-free survival in patients with T-NHL (median OS 17.0 vs. 7.0 months; *p* = 0.012). These results are consistent with a previous report which describes that RBMX controls myeloid leukemogenesis by regulating the chromatin state [21]. As a caveat, all patients with high RBMX expression died within 58 months after initial diagnosis. Continuously, the multivariable analysis has been showing RBMX to be an independent prognostic marker for overall survival (*p* = 0.007; HR; 0.204; 95% CI: 0.064–0.646) and progression-free survival (*p* = 0.006; HR = 0.235; 95% CI: 0.083–0.666) in T-NHL. In patients with nodal T-NHL phenotype, the RBMX expression was an independent prognostic marker solely for overall survival (*p* = 0.038; HR = 0.149; 95% CI: 0.025–0.898). As shown above, our results are not significant in the smaller subgroup of primary nodal T-NHL; this could be due to the small size of the subgroup (*n* = 34).

Our results suggest that RBMX expression contributes to chemotherapy resistance and thus affects prognosis in patients with T-NHL. These findings are consistent with previous mentioned studies. However, the current results are subject to several limitations mainly caused by the retrospective character of this analysis and the high heterogeneity in the investigated cohort. Furthermore, the small sample size increases the risk of statistical errors. Therefore, studies with larger cohorts might provide a better understanding on the function of RBMX during carcinogenesis and chemotherapy resistance. Moreover, further cellular experiments are needed to elucidate this issue. In conclusion, our study found that low RBMX protein expression predicted better response to anthracycline-containing first-line treatment, overall survival, and progression-free survival in patients with T-cell non-Hodgkin’s lymphomas. Hence, RBMX may be a potential therapeutic target and prognostic marker in T-cell lymphoma.

## 5. Conclusions

In conclusion, this study showed that low RBMX expression was associated with better response to anthracycline-containing first-line treatment and an improved overall survival (OS) and progression-free survival (PFS) in patients with T-cell non-Hodgkin’s lymphomas.

## Figures and Tables

**Table 1:** Characteristics of patients.

Characteristic		T-NHL *n* = 43	Nodal T-NHL *n* = 34
		*n* (%)	*n* (%)
Sex	Female	14 (33)	12 (35)
	Male	29 (67)	22 (65)
Age (years)	<65	18 (42)	14 (41)
	≥65	25 (58)	20 (59)
B Symptoms	Absent	24 (56)	20 (59)
	Present	19 (44)	14 (41)
Bone marrow involvement	Absent	29 (67)	25 (74)
	Present	10 (23)	7 (21)
	Not evaluable	4 (9)	2 (6)
Ann Arbor stage	Stages I and II	8 (19)	6 (18)
	Stages II and IV	28 (65)	25 (74)
	Not evaluable	7 (16)	3 (9)
IPI	0–2	18 (42)	15 (44)
	2–4	19 (44)	17 (50)
	Not evaluable	6 (14)	2 (6)
ECOG	0–1	19 (44)	16 (47)
	2–5	5 (12)	5 (15)
	Not evaluable	19 (44)	13 (38)
WBC	Normal	12 (28)	8 (24)
	Upper limit of normal	11 (26)	7 (21)
	Not evaluable	20 (47)	19 (56)
LDH	Normal	6 (14)	2 (6)
	Upper limit of normal	18 (42)	17 (50)
	Not evaluable	19 (44)	15 (44)
Ki-67 expression	<65%	15 (35)	12 (35)
	≥65%	13 (30)	10 (29)
	Not evaluable	15 (35)	12 (35)
Relapse	Absent	21 (49)	14 (41)
	Present	22 (51)	20 (59)
First-line treatment	R-CHO(E)P	34 (79)	33 (97)
	Others	9 (21)	1 (1)

Abbreviations: T-NHL: T-cell non-Hodgkin’s lymphoma; *n*: number; IPI: International Prognostic Index, ECOG: Eastern Cooperative Oncology Group status; WBC: white blood cell count; LDH: lactate dehydrogenase level.

**Table 2:** Correlations between RBMX expression (H-score) and the clinicopathological characteristics in T-NHL and nodal T-NHL.

Characteristic		T-NHL		Nodal T-NHL	
		RBMX Expression (Mean)	*p*-Value	RBMX Expression (Mean)	*p*-Value
Sex	Female	131	0.524	132	0.544
	Male	146		146	
Age (years)	<65	155	0.310	154	0.308
	≥65	132		131	
B Symptoms	Absent	133	0.570	132	0.328
	Present	146		154	
Bone marrow involvement	Absent	152	0.090	153	0.034
	Present	107		94	
Ann Arbor stage	Stages I and II	153	0.402	158	0.494
	Stages II and IV	130		137	
IPI	0–2	140	0.433	154	0.295
	2–4	122		129	
ECOG	0–1	140	0.726	145	0.634
	2–5	128		128	
WBC	Normal	193	0.023	186	0.089
	Upper limit of normal	118		126	
LDH	Normal	126	0.741	195	0.304
	Upper limit of normal	137		144	
Ki-67 expression	<65%	130	0.375	154	0.493
	≥65%	155		135	
Relapse	Absent	145	0.762	148	0.603
	Present	139		136	
Response to first-line treatment	Resistant	178	0.029	167	0.119
	Sensitive	128		130	

Abbreviations: T-NHL: T-cell non-Hodgkin’s lymphoma; IPI: International Prognostic Index; ECOG: Eastern Cooperative Oncology Group status; WBC: white blood cell count; LDH: lactate dehydrogenase level.

**Table 3:** Multivariable analysis of overall survival (OS) and progression-free survival (PFS) in T-NHL and nodal T-NHL.

Multivariable Analysis		Overall Survival				Progression-Free Survival			
Variable	Categories	HR	95% CI		*p*-Value	HR	95% CI		*p*-Value
			LL	UL			LL	UL	
T-NHL cohort (*n* = 39)									
Sex	female vs. male	1.996	0.618	6.445	0.248	1.329	0.536	3.295	0.539
Age in years		1.046	0.986	1.110	0.138	1.002	0.956	1.051	0.924
B Symptoms	absent vs. present	0.592	0.179	1.957	0.390	0.466	0.194	1.118	0.087
Bone marrow involvement	absent vs. present	0.623	0.192	2.020	0.431	0.243	0.094	0.628	0.004
RBMX expression	low vs. high	0.204	0.064	0.646	0.007	0.235	0.083	0.666	0.006
Nodal T-NHL cohort (*n* = 34)									
Sex	female vs. male	1.777	0.376	8.402	0.469	0.796	0.258	2.455	0.691
Age in years		1.026	0.964	1.093	0.416	0.989	0.937	1.043	0.68
Ann Arbor stage	I-II vs. III-IV	5.461	0.945	3.155	0.058	0.928	0.248	3.482	0.912
B Symptoms	absent vs. present	0.365	0.056	2.371	0.291	0.606	0.180	2.038	0.419
Bone marrow involvement	absent vs. present	0.497	0.072	3.414	0.477	0.248	0.080	0.771	0.016
RBMX expression	low vs. high	0.149	0.025	0.898	0.038	0.361	0.107	1.221	0.101

Abbreviations: HR: hazard ratio; CI: confidence interval; LL: lower limit; UL: upper limit; *n*: number; RBMX low: RBMX expression with an H-score below 175; RBMX high: RBMX expression above or equal to an H-score of 175.

## Data Availability

All relevant data are within the paper.
